# **Prevalence of molar incisor hypomineralisation and associated factors** **amongst** **8-year-olds in Ireland**

**DOI:** 10.1007/s40368-025-01033-6

**Published:** 2025-04-28

**Authors:** S. Nic Ghearailt, N. Coffey, M. Harding, H. Whelton, M. Cronin, P. James

**Affiliations:** 1 Cork University Dental School and Hospital, University College Cork, Cork, T12 E8YV Ireland; 2 Health Service Executive, Dental Services, Arklow Primary Care Centre, Castle Park, Arklow, Co. Wicklow Y14 AH57 Ireland; 3 Royal College of Surgeons in Ireland, 123 St Stephen’s Green, Dublin, D02 YN77 Ireland; 4 Oral Health Services Research Centre, University College Cork, Cork, T12 E9YV Ireland; 5 College of Medicine and Health, University College Cork, Cork, T12 EDK0 Ireland; 6 School of Mathematical Sciences, University College Cork, Cork, T12 XF62 Ireland

**Keywords:** MIH, Molar incisor hypomineralisation, Prevalence, Dental caries, Oral health-related quality of life, Water fluoridation

## Abstract

**Purpose:**

To describe the prevalence of molar incisor hypomineralisation (MIH) and a selection of potentially associated factors amongst 8-year-olds in Ireland.

**Methods:**

This study reports cross-sectional data from the Fluoride and Caring for Children’s Teeth (FACCT) study 2017. Eight-year-olds in Dublin (*n* = 786) and Cork-Kerry (*n* = 1524) were clinically examined for MIH (EAPD criteria) and dental caries (DMFT). The association between potential aetiological factors and MIH was assessed using multivariable logistic regression. Potential effects of MIH on dental caries, oral health-related quality of life (OHRQoL) and parental perceptions of the appearance of their child’s permanent incisors were investigated.

**Results:**

MIH prevalence was 11% in Dublin and 9% in Cork-Kerry. In Dublin, prevalence of MIH was higher amongst children who had health problems in the past year. There was no association between community water fluoridation and MIH in Cork-Kerry. In both regions, dental caries was higher amongst children with MIH. Parents of children with MIH in Dublin and Cork-Kerry were more likely to have noticed marks on their child’s permanent incisors. In Dublin, MIH was associated with poorer parent-reported OHRQoL. Parents of children with MIH in Cork-Kerry were less likely to be satisfied with the colour of their child’s permanent incisors.

**Conclusions:**

MIH prevalence was 9–11%. The association between MIH and dental caries suggests that MIH severity in Ireland may be high. There is an urgent need for national data on MIH prevalence and severity, early identification of children with MIH and provision of preventive and treatment services in line with international best practise.

**Supplementary Information:**

The online version contains supplementary material available at 10.1007/s40368-025-01033-6.

## Introduction

Molar incisor hypomineralisation (MIH) is a developmental defect of enamel, characterised by demarcated opacities that affect one or more first permanent molars, with or without involvement of the permanent incisors (Weerheijm et al. 2001). The degree of hypomineralization can vary and may be accompanied by post-eruptive enamel breakdown (Weerheijm 2003a). As a result, affected individuals often experience plaque accumulation and difficulties with oral hygiene due to sensitivity, which can increase the risk of dental caries development (Weerheijm 2003a). Children with MIH experience higher levels of dental caries (Americano et al. 2017) and may require a range of dental care, from preventive measures to restorative procedures and possibly extractions (Lygidakis et al. 2010). In addition, due to hypersensitivity, affected teeth can be difficult to anaesthetise (Rodd et al. 2007; Somani et al. 2022). MIH significantly impacts the oral health-related quality of life (OHRQoL) of affected children (Amrollahi et al. 2023; Jälevik et al. 2022).

A systematic review and meta-analysis of the estimated global, super-regional, regional, and national burden of MIH prevalence yielded a mean (95% CI) of 12.9% (11.7, 14.3), with significant differences between super-regions, regions, and countries (Schwendicke et al. 2019; Schwendicke et al. 2018). This review included 99 studies from 43 countries and estimated that over 800-million people had MIH in 2016. Another systematic review, which included 116 observational studies from 50 different countries, estimated that the prevalence of MIH was 13.5%, with 36.3% of cases being classified as moderate to severe (Lopes et al. 2021). Zhao et al. (2018) reported a similar estimate of pooled global prevalence of 14.2% (12.6, 15.8), with a wide range of prevalence estimates across the 70 included studies (0.5–40.2%).

The available evidence suggests a multifactorial aetiology for MIH (Lygidakis et al. 2022). Peri- and postnatal aetiological risk factors are considered most relevant to the development of MIH (Bussaneli et al. 2022; Garot et al. 2022). Perinatal hypoxia, caesarean section and preterm birth are associated with MIH (Garot et al. 2022). Amongst the postnatal factors explored, early childhood illnesses including measles, urinary tract infection, otitis media, gastric disorders and kidney diseases are implicated in the aetiology of MIH (Garot et al. 2022). Systematic reviews have reported an association between MIH and respiratory conditions such as asthma and pneumonia during early childhood (Fatturi et al. 2019; Garot et al. 2022; Silva et al. 2016). In addition, episodes of fever in early childhood have been linked with MIH. However, it remains unclear whether MIH is related to the fever itself, the underlying childhood illness that caused the fever, or the antibiotics used to treat the illness (Garot 2022). The impact of systemic aetiological factors on the clinical presentation and severity of MIH may be influenced by the duration, strength and timing of their action on amelogenesis (Lygidakis et al. 2022). Furthermore, it is likely that the effect of systemic risk factors is mediated by genetic and/or epigenetic factors (Lygidakis et al. 2022; Vieira and Manton 2019). These aetiological complexities may account for the variable and asymmetrical clinical presentations of MIH.

Investigations into the aetiology of MIH have included other potentially associated factors. For example, the relationship between socioeconomic status (SES) and MIH has been investigated on the basis that lower SES may be associated with poorer health in childhood. However, the available evidence does not consistently support an association between low SES and MIH (Balmer et al. 2012; Ghanim et al. 2014; Harz et al. 2023). In addition, it has been proposed that fluoride could play a role in the pathogenesis of MIH, by strengthening enamel and reducing MIH or conversely, by increasing the risk of MIH (Balmer et al. 2015; Fernandes et al. 2021; Veneri et al. 2024). However, whilst exposure to fluoride is associated with the development of diffuse enamel opacities (Clarkson and O’Mullane 1992; Cutress et al. 1985), most studies have found no link between exposure to fluoride and demarcated enamel opacities or MIH (Crombie et al. 2009; Veneri et al. 2024).

MIH may have a significant negative effect on the oral health and wellbeing of affected individuals. A recent systematic review by Gevert et al. (2024) reported that the most common clinical consequences of MIH are caries lesions, hypersensitivity, and post-eruptive breakdown. Individuals with MIH may be at increased risk of developing dental caries due to plaque retention on tooth surfaces affected by post-eruptive enamel breakdown, a situation which is complicated by difficulty maintaining adequate oral hygiene on hypersensitive teeth (Weerheijm 2003a). In addition, MIH may negatively impact children’s OHRQoL particularly concerning the domains “oral symptoms”, “functional limitations” and “emotional well-being” (Amrollahi et al. 2023; Jälevik et al. 2022). MIH affecting the permanent incisors may have a negative impact on aesthetics as perceived by affected children and their parents (Fragelli et al. 2021; Leal et al. 2017). Furthermore, young people may make negative value judgements about the dental appearance of other young people with visible enamel defects (Craig et al. 2015).

There are no published data on the prevalence of MIH or its associated factors in the Republic of Ireland. This study aims to describe the prevalence of MIH and a selection of potentially associated factors amongst 8-year-old school children in two regions of the Republic of Ireland.

## Methods

The study was performed in accordance with the ethical standards as laid out in the 1964 Declaration of Helsinki and ethical approval was obtained from the Clinical Research Ethics Committee of the Cork Teaching Hospitals, University College Cork, Ireland (ECM 5 (2) 07/05/13 and ECM 3 (e) 05/07/16). Written informed consent from parents/guardians, and child assent were obtained. This study adheres to the Strengthening the Reporting of Observational Studies in Epidemiology (STROBE) guidelines (von Elm et al. 2008) (Online Resource 1).

### Study design

This study reports on the analysis of cross-sectional data for 8-year-olds who participated in the FACCT study in 2017. Children were recruited to the longitudinal component of the FACCT study when they were 5-year-olds in 2014 (phase 1) and were followed up at age 8 in 2017 (phase 2). The data reported in this study were collected in phase 2 of the study, in 2017. Online Resource 2 indicates where the MIH study fits within the FACCT study.

### Study location

The study was conducted in randomly selected primary schools in counties Dublin, Cork and Kerry in the Republic of Ireland. Dublin is a metropolis, where 28% of the Irish population resided at the time of the study (Central Statistics Office 2017). Counties Cork and Kerry include a city, country towns and rural areas. Drinking water supplies in Dublin are fluoridated at 0.7-ppm fluoride (Government of Ireland 2007), whereas counties Cork and Kerry have a mix of areas with and without community water fluoridation.

### Study sample

Participants were selected at the commencement of the FACCT study in 2014 to provide a representative sample of children with and without exposure to community water fluoridation as described in the FACCT clinical fieldwork protocol (James et al. 2018). The FACCT sample in 2014 was selected using multistage stratified cluster random sampling with the school as the primary sampling unit. The selection of participants within classes was random (computer generated). Adjustments to sample size accounted for the design effect due to using a cluster sampling technique and loss to follow-up. The sample for the current study comprised children who participated in phase 1 of the FACCT study when they were in junior infants (aged 5 years), who were followed up and examined for MIH in phase 2 of FACCT when they were in second class (aged 8 years).

### Parental-caregiver and child-completed questionnaires

Parental-caregiver completed questionnaires were used to collect information about explanatory variables including the child’s age and gender, and parental socio-economic details (ownership of a means tested Medical Card and highest level of education attained by the parents). Parents were asked questions regarding their perceptions of their child’s permanent incisors, including whether they were happy with the colour of the teeth and whether they had noticed any brown, creamy or white marks on the teeth that do not rub off. Parents were also asked about their child’s general health in the past year.

Parental perceptions of child OHRQoL were measured using the short form of the Parental-Caregivers Perceptions Questionnaire (P-CPQ) (Jokovic et al. 2003). Child-reported oral health-related quality of life (COHRQoL) was measured using the child perceptions questionnaire for 8–10-year-olds (CPQ_8–10_ [long form]) (Jokovic et al. 2004). Children completed the questionnaire on a secure laptop, whilst supervised by an adult. Paper and Irish language versions were available on request.

### Exposure to community water fluoridation

The FACCT study included representative samples of children with different levels of exposure to community water fluoridation at 0.7-ppm. Measurement of exposure to community water fluoridation was described elsewhere (James et al. 2021, 2018). Each child’s lifetime exposure to community water fluoridation was categorised based on the fluoride concentration of water supplying their home address and all previous addresses. Fluoridation status was classified as lifetime exposure to community water fluoridation (Full-CWF), sporadic exposure to community water fluoridation (Part-CWF), no exposure to community water fluoridation (No-CWF) and unknown exposure to community water fluoridation (Unknown).

### Clinical examinations

The examining teams, comprised of dentists assisted by dental nurses, were trained to measure dental caries (cavitated and non-cavitated (visual) dentine level, D_3vc_MFT), fluorosis and developmental defects of enamel (including MIH) in advance of the fieldwork by experienced benchmark examiners. Training encompassed a combination of small group interactive teaching sessions, individual scoring and discussion of photographs of dental caries, fluorosis and developmental defects of enamel and a school-based clinical training session. Dental examiners were trained to distinguish between fluoride related (diffuse) and non-fluoride related (demarcated) enamel opacities. Calibration examinations were carried out for measurement of dental caries but there was no calibration for measurement of MIH. Fieldwork manuals were produced to assist examining teams in organising and conducting oral epidemiological fieldwork (James et al. 2018).

#### Measurement of MIH

The case definition and identification of MIH was according to the European Association of Paediatric Dentistry (EAPD) judgement criteria (Weerheijm et al. 2003b). All erupted first permanent molars and incisors were examined for signs of MIH, with the child supine, using a portable dental light (‘Daray’ lamp) for illumination. First permanent molars and incisors were examined for MIH if any part of the surface had penetrated the oral mucosa. A partially erupted tooth was judged to be normal unless there was a defect on the erupted portion of the tooth**.** The teeth were examined wet, and a cotton wool roll used, if necessary, to clean the teeth prior to the examination. MIH was judged to be present (yes) or absent (no).

The following criteria were taken into consideration when making the judgement regarding the presence or absence of MIH:

- absence or presence of demarcated enamel opacities;
- post-eruptive enamel breakdown;
- atypical restorations;
- extraction of molars due to MIH.

Presence of MIH was recorded if one or more first permanent molars met the criteria listed above. Permanent incisors did not need to be affected for a judgement of MIH to be recorded.

#### Measurement of dental caries

The method for measuring dental caries in permanent teeth of 8-year-olds was described previously (James et al. 2018). Teeth were examined wet with the child supine and lighting provided by a portable dental light (‘Daray’ lamp). Dental caries was recorded for permanent teeth using an extended version of the World Health Organisation (WHO) criteria (World Health Organisation 2013) at cavitated and non-cavitated (visual) dentine level (D_3vc_MFT)(Whelton et al. 2006). A comprehensive periodontal evaluation (CPE) probe was used to remove food debris or to confirm cavitation. During the 2017 fieldwork, kappa (κ) scores for inter examiner agreement (with benchmark examiners) ranged from 0.55 to 0.92 (median 0.81), whilst intra examiner κ scores ranged from 0.77 to 1.00 (median 0.92) (James et al. 2021).

### Statistical analysis

A summary of the data analysis methods is presented in Table 1. The association between the potential aetiological factors (economic disadvantage, fluoridation status, mother’s education, father’s education and the child’s general health) and MIH was assessed using multivariable logistic regression adjusted for gender and age. Fluoridation status was not included for Dublin as the sample was predominately fully fluoridated. The association between MIH as an explanatory variable and the outcomes (1) dental caries, (2) CPQ_8-10_ score, (3) P-CPQ score, (4) whether parents were happy with the colour of their child’s ‘permanent (adult) front teeth’, (5) whether parents had noticed any brown, creamy or white marks on the child’s ‘permanent (adult) front teeth’ that do not rub off were analysed using Poisson regression, Analysis of Variance (ANOVA) or Chi-square tests as appropriate (Table 1). These outcome variables represented potential effects of MIH. If a child had missing data for a particular variable, the child was not included in the analysis that involved that variable; missing data were not imputed. Separate analyses were performed for Dublin and Cork-Kerry. Odds ratios, ratio of means or difference in means were calculated based on multivariable logistic regression, Poisson regression, ANOVA and Chi-square tests and reported with 95% confidence intervals (95% CI). A 5% level of significance was used in all statistical tests. The software used was SAS® (Version 9.4).

**Table 1:** Summary of data analysis methods

Explanatory variables	Outcome variables	Statistical method
Gender, age, medical card, fluoridation status (Cork-Kerry only), mother’s education, father’s education, child healthy (no problems)	MIH (yes/no)	Multivariable Logistic Regression
MIH (yes/no)	D_3vc_MFT	Poisson Regression
MIH (yes/no)	CPQ_8-10_ score	Analysis of Variance (ANOVA)
MIH (yes/no)	P-CPQ score	Analysis of Variance (ANOVA)
MIH (yes/no)	Parent happy with the colour of the child’s permanent incisors	Chi-square test
MIH (yes/no)	Whether the parent has noticed marks on the child’s permanent incisors	Chi-square test

## Results

**Fig. 1:**
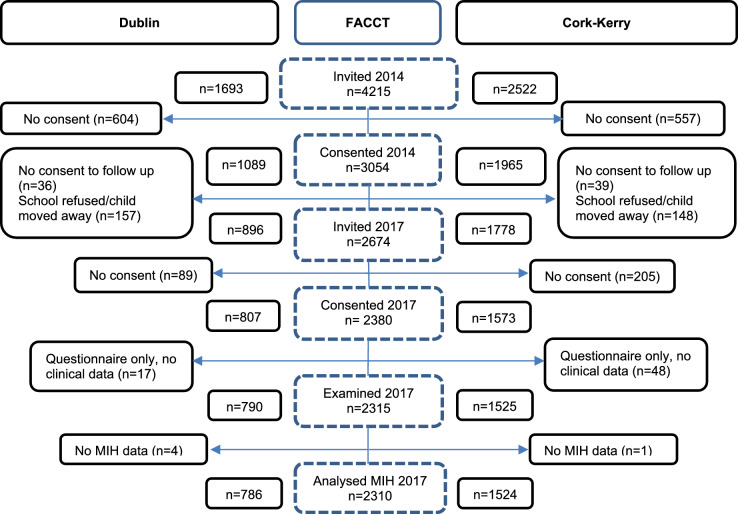
Flow of participants through the FACCT study showing numbers included in MIH analyses

A flowchart showing the numbers of children at each stage of the study is presented in Fig. 1. The response rate was 72% (3054/4215) in phase 1 of the study in 2014. In 2017 (phase 2), 55% (2310/4215) of those invited to participate in the study in 2014 were followed up and examined for MIH. There was an even balance of gender amongst the children examined in Dublin and Cork-Kerry in 2017. The proportion female was 53% in Dublin and 52% in Cork-Kerry. Mean age was similar in Dublin (8.2; SD 0.4) and Cork-Kerry (8.4; SD 0.4). The proportion of children who were dependents of Medical Card holders was 29% in Dublin and 25% in Cork-Kerry.

### Prevalence of MIH

The prevalence of MIH for 8-year-old children was 11% (85/786; 95% CI: 9, 13) in Dublin and 9% (144/1524; 95% CI: 8, 11) in Cork-Kerry (Table 2).

**Table 2:** Prevalence of MIH, demographics and potential aetiological factors for MIH amongst 8-year-olds in Dublin and Cork-Kerry

	Dublin (*n* = 786) MIH		Cork-Kerry (*n* = 1524) MIH	
	Yes	No	Yes	No
	*n* (%)	*n* (%)	*n* (%)	*n* (%)
Numbers Examined	85 (11)	701 (89)	144 (9)	1380 (91)
Demographics and Potential Aetiological Factors				
Gender^a^				
Female	40 (10)	374 (90)	78 (10)	716 (90)
Male	45 (12)	327 (88)	66 (9)	664 (91)
Age^a^ (Mean (SD))	8.2 (0.4)	8.2 (0.3)	8.4 (0.4)	8.4 (0.4)
Economic disadvantage^b^				
Medical Card	26 (12)	199 (88)	21 (6)	354 (94)
No Medical Card	59 (11)	487 (89)	122 (11)	998 (89)
Missing	0	15	1	28
Fluoridation Status^a^				
Full-CWF	80 (11)	623 (89)	32 (9)	344 (91)
Part-CWF	0 (0)	12 (100)	29 (9)	282 (91)
No-CWF	0 (0)	1 (100)	75 (10)	696 (90)
Unknown	5 (7)	65 (93)	8 (12)	58 (88)
Mother’s education				
Leaving Certificate or less^c^	27 (13)	178 (87)	39 (11)	322 (89)
Diploma/Certificate or higher	57 (10)	495 (90)	100 (9)	1011 (91)
Missing	1	28	5	47
Father’s education				
Leaving Certificate or less	27 (13)	181 (87)	48 (9)	466 (91)
Diploma/Certificate or higher	52 (11)	411 (89)	86 (10)	800 (90)
Missing	6	109	10	114
Child very healthy, no problems				
Yes	41 (8)	482 (92)	101 (10)	955 (90)
No	44 (18)	207 (82)	42 (10)	398 (90)
Missing	0	12	1	0

^a^No missing data, ^b^Medical Card ownership indicates socio-economic disadvantage, ^c^Attainment of Leaving Certificate indicates completion of second level education, *SD* Standard Deviation

#### Potential aetiological factors for MIH

Demographics and participant characteristics representing potential aetiological factors for MIH amongst children in Dublin and Cork-Kerry are presented in Table 2. The results of multivariable logistic regression of the association between the potential aetiological factors and MIH are summarised in Table 3. In Dublin, multivariable analysis indicated no association between Medical Card ownership or parental education and MIH. However, in Cork-Kerry the prevalence of MIH was lower amongst those who had a Medical Card (6%) compared to those without a Medical Card (11%). After controlling for the effects of other explanatory variables, the odds of having MIH were 56% lower for those with a Medical Card than for those without (OR 0.44; 95% CI: 0.26, 0.75; *p* = 0.003). In Cork-Kerry the prevalence of MIH was higher amongst children whose mothers had completed second level education or less (11%) compared to those whose mothers had a higher level of education (9%). The difference was statistically significant after controlling for the effects of other explanatory variables (OR 1.65; 95% CI: 1.04, 2.63; *p* = 0.034). There was no association between exposure to community water fluoridation and MIH in Cork-Kerry. 

In Dublin the prevalence of MIH was higher amongst children who had health problems in the past year (18%) compared with those who were healthy and did not report having any problems in the past year (8%). After controlling for the effects of other explanatory variables, the difference was statistically significant (OR 2.57; 95% CI 1.57, 4.21; *p* < 0.001). In Cork/Kerry, no such association was found (*p* = 0.466).

**Table 3:** Results of multivariable logistic regression analyses of potential aetiological factors for MIH amongst 8-year-olds in Dublin and Cork-Kerry

Demographics and Potential Aetiological Factors	Dublin		Cork-Kerry	
	OR (95% CI)	*p *value	OR (95% CI)	*p* value
Gender				
Female	0.80 (0.49, 1.29)	0.361	1.07 (0.74, 1.53)	0.730
Male	Reference		Reference	
Age	1.02 (0.51, 2.07)	0.949	1.33 (0.80, 2.20)	0.265
Economic disadvantage^a^				
Medical Card yes	0.90 (0.49, 1.65)	0.735	0.44 (0.26, 0.75)	0.003*
Medical Card no	Reference		Reference	
Fluoridation status				
Full-CWF			Reference	
Part-CWF	N/A		1.04 (0.60, 1.81)	0.887
No-CWF	N/A		1.03 (0.65, 1.63)	0.889
Unknown	N/A		1.66 (0.72, 3.87)	0.237
Mother’s education				
Leaving Certificate or less^b^	1.22 (0.65, 2.28)	0.545	1.65 (1.04, 2.63)	0.034*
Diploma/Certificate or higher	Reference		Reference	
Father’s education				
Leaving Certificate or less	1.03 (0.56, 1.90)	0.928	0.89 (0.58, 1.37)	0.593
Diploma/Certificate or higher	Reference		Reference	
Child very healthy, no problems				
Yes	Reference	< 0.001*	Reference	0.466
No	2.57 (1.57, 4.21)		1.16 (0.78, 1.73)	

^a^Medical card ownership indicates socio-economic disadvantage, ^b^Attainment of Leaving Certificate indicates completion of second level education, *p < 0.05, *OR* Odds Ratio (adjusted), *CI* Confidence Interval, *N/A* Not Applicable, Unadjusted Odds Ratios are presented in Online Resource 3

#### Potential effects of MIH

The distribution of outcome variables representing potential effects of MIH amongst children in Dublin and Cork-Kerry are reported in Table 4. Results of univariate analyses of the association between MIH and the outcome variables are summarised in Table 5.

**Table 4:** Distribution of outcome variables representing potential effects of MIH amongst 8-year-olds in Dublin and Cork-Kerry

	Dublin *(n* = 786) MIH		Cork-Kerry (*n* = 1524) MIH	
	Yes	No	Yes	No
	*n* (%)	*n* (%)	*n* (%)	*n* (%)
Numbers Examined	85 (11)	701 (89)	144 (9)	1380 (91)
Potential Effects of MIH				
Parent happy with colour of child’s permanent incisors				
Yes	48 (58)	435 (64)	88 (62)	938 (70)
No	30 (36)	181 (27)	47 (33)	311 (23)
Don’t Know	5 (6)	64 (9)	7 (5)	98 (7)
Missing	2	21	2	33
Parent noticed marks on child's permanent incisors				
Yes	33 (40)	170 (25)	55 (38)	309 (23)
No	46 (55)	470 (69)	84 (59)	981 (73)
Don’t Know	4 (5)	39 (6)	4 (3)	56 (4)
Missing	2	22	1	34

	Mean (SD)	Mean (SD)	Mean (SD)	Mean (SD)
D_3vc_MFT	0.7 (1.1)	0.2 (0.6)	0.8 (1.1)	0.3 (0.8)
CPQ_8-10_ score	13.5 (11.1)	12.1 (12.1)	11.1 (12.1)	10.4 (11.0)
P-CPQ score	5.5 (5.0)	4.1 (4.9)	3.8 (4.5)	3.6 (4.4)

D_3vc_MFT includes dentinal caries at cavitated and non-cavitated (visual) level, SD Standard deviation, *CPQ*_*8-10*_ Child Perceptions Questionnaire for 8-10-year-olds, *P-CPQ *Parental Caregivers Questionnaire, Missing data for D_3vc_MFT included one child from Cork-Kerry (No MIH), Missing data for CPQ_8-10_ included 26 children from Dublin (3 MIH, 23 No MIH) and 40 children from Cork-Kerry (1 MIH, 39 No MIH), Missing data for P-CPQ included 25 children in Dublin (No MIH) and 31 children in Cork-Kerry (No MIH)

**Table 5:** Results of univariate analysis of the association between MIH and outcome variables representing potential effects of MIH amongst 8-year-olds in Dublin and Cork-Kerry

Potential Effects of MIH	Dublin		Cork-Kerry	
	Mean Ratio (95% CI)	*p* value	Mean Ratio (95% CI)	*p* value
D_3vc_MFT^a^	3.58 (2.67, 4.82)	< 0.001*	2.87 (2.34, 3.51)	< 0.001*

	Difference in means (95% CI)	*p* value	Difference in means (95% CI)	*p* value
CPQ_8-10_ score^b^	1.4 (− 1.3, 4.2)	0.315	0.8 (− 1.2, 2.7)	0.440
P-CPQ score^b^	1.4 (0.3, 2.6)	0.010*	0.2 (− 0.5, 1.0)	0.546

	Odds Ratio (95% CI)	*p* value	Odds Ratio (95% CI)	*p* value
Parent happy with colour of child’s permanent incisors^c^	0.67 (0.41, 1.08)	0.149	0.62 (0.43, 0.90)	0.024*
Parent noticed marks on child's permanent incisors^c^	1.98 (1.23, 3.21)	0.017*	2.08 (1.45, 2.99)	< 0.001*

^a^Poisson regression, ^b^ANOVA Analysis of Variance, ^c^Chi-square test, D_3vc_MFT includes dentinal caries at cavitated and non-cavitated (visual) level, *CPQ*_*8-10*_ Child Perceptions Questionnaire for 8-10-year-olds, *P-CPQ* Parental Caregivers Questionnaire, *p < 0.05, *CI* Confidence Interval

#### Dental caries

In Dublin and Cork-Kerry, dental caries was higher amongst children with MIH than amongst those without MIH. The mean D_3vc_MFT for children with MIH in Dublin was 0.7 (SD 1.1) and for those without MIH was 0.2 (SD 0.6) (ratio of means = 3.58; 95% CI: 2.67, 4.82; *p* < 0.001). The mean D_3vc_MFT for children with MIH in Cork-Kerry was 0.8 (SD 1.1) and for those without MIH was 0.3 (SD 0.8) (ratio of means = 2.87; 95% CI: 2.34, 3.51; *p* < 0.001).

#### Oral health-related quality of life (OHRQoL)

There was no association between MIH and OHRQoL (child or parent reported), except in Dublin, where the mean P-CPQ score was higher amongst parents/caregivers of children with MIH (indicating poorer OHRQoL) (difference in means = 1.4; 95% CI: 0.3, 2.6; *p* = 0.010).

#### Parental-Caregiver’s perception of child’s teeth

In Dublin, there was no association between MIH and the parent’s satisfaction with the colour of their child’s permanent incisors. However, in Cork-Kerry, parents of children with MIH were less likely to be happy with the colour of their child’s permanent incisors than parents of children without MIH (62% vs. 70%; OR 0.62; 95% CI: 0.43, 0.90; *p* = 0.024).

There was a significant association in both regions between MIH and the parent noticing marks on the child’s permanent incisors. In Dublin, amongst children with MIH, a higher proportion of parents reported noticing marks on the permanent incisors than parents of children without MIH (40% vs. 25%; OR1.98, 95% CI: 1.23, 3.21; *p* = 0.017). Results were similar in Cork-Kerry (38% vs. 23%; OR 2.08, 95% CI: 1.45, 2.99; *p* < 0.001).

## Discussion

The prevalence of MIH was 11% in Dublin and 9% in Cork-Kerry in the Republic of Ireland. These figures are generally consistent with international studies involving similar age groups. The pooled global prevalence of MIH is estimated to be between 12.9% and 14.2% (Lopes et al. 2021; Schwendicke et al. 2019; Schwendicke et al. 2018; Zhao et al. 2018). There were regional differences amongst many of the factors associated with MIH. Analysis of the association between measures of SES and MIH produced conflicting results. In Dublin, prevalence of MIH was higher amongst children who had health problems in the past year. In Cork-Kerry, which included a mix of urban and rural areas with and without community water fluoridation, there was no association between exposure to community water fluoridation and MIH. MIH was associated with higher dental caries levels in both regions. Furthermore, in Dublin and Cork-Kerry, parents of children with MIH were more likely to have noticed marks on the child’s permanent incisors. In Dublin, there was an association between MIH and poorer parent-reported OHRQoL. Lastly, in Cork-Kerry, parents of children with MIH were less likely to be satisfied with the colour of the child’s permanent incisors.

In Dublin, no association was found between MIH and Medical Card ownership or parental education. However, in Cork-Kerry, the odds of MIH were lower for Medical Card holders. Conversely, the odds of MIH were higher amongst children whose mothers had attained the Leaving Certificate or less compared with those whose mothers had attained a higher level of education. Using Medical Card ownership as a proxy measure for SES has its limitations, which may help explain the contradictory findings in the current study. In Ireland, Medical Card ownership does not always accurately reflect economic disadvantage, as some middle-income families may qualify due to specific health needs. Furthermore, eligibility for a Medical Card is means-tested and can change over time. Consequently, in this study, parental education may serve as a more reliable indicator of the child’s SES. Inconsistent findings regarding an association between measures of SES and MIH exist in the literature. Some studies have reported the prevalence of MIH to be higher amongst those with higher SES (Balmer et al. 2012; Biondi et al. 2011; Teixeira et al. 2018). Others have reported an association with lower SES (Harz et al. 2023), or have found no association (Ghanim et al. 2014; Mahoney and Morrison 2011; Reis et al. 2021).

In Dublin, children with MIH were more likely to have had health problems within the past year than those without MIH. However, this association was not observed in the more rural area of Cork-Kerry. There is a clear connexion between early childhood illnesses, such as respiratory conditions, and MIH (Fatturi et al. 2019; Garot et al. 2022; Silva et al. 2016). Experiencing health problems in the past year may reflect poorer overall health during childhood, which could explain the association with MIH amongst children in Dublin. The difference in results between the Dublin and Cork-Kerry regions warrants further investigation. The aetiology of MIH involves a complex interaction of systemic, environmental, and genetic factors (Feltrin-Souza et al. 2024). It is plausible that environmental influences, such as exposure to air pollutants in the Dublin metropolitan area may have contributed to poorer general health and greater risk of developing MIH.

Based on a large representative sample of children with meticulous, individual level classification of exposure to water fluoridation, we found no association between community water fluoridation and MIH. The association between fluoride exposure through water fluoridation and increased prevalence of diffuse enamel opacities rather than demarcated enamel opacities is well established (Clarkson and O’Mullane 1992; Cutress et al. 1985). Whilst it is accepted that fluoride is unlikely to play a major role in the aetiology of MIH (Crombie et al. 2009), it has been proposed that fluoride could influence the development of MIH, either reducing or increasing the risk of MIH (Balmer et al. 2015; Fernandes et al. 2021; Veneri et al. 2024). However, systematic reviews have reported no association between fluoride exposure (predominantly water fluoridation) and demarcated opacities or MIH (Crombie et al. 2009; Veneri et al. 2024). Whilst the systematic review by Veneri et al. (2024) suggested a dose–response relationship, with a decreased risk of MIH up to a water fluoride concentration of 1-ppm, and an increased risk of MIH at higher water fluoride concentrations, this finding was heavily influenced by the results of one of the 13 included studies (Angelillo et al. 1990), conducted prior to the identification of MIH as a distinct condition, and an outlier relative to the results of the other included studies. The results of our study are in alignment with most of the available evidence in indicating that there is no association between community water fluoridation and MIH.

MIH was associated with dental caries in the newly erupted first permanent molars of 8-year-olds in both geographical regions. The findings are consistent with a systematic review of the association between MIH and dental caries (Americano et al. 2017). This suggests that there may be a substantial treatment need associated with prevalent cases of MIH in Ireland and that early identification of MIH for preventive intervention is crucial. The EAPD emphasise the importance of establishing early, evidence-based caries prevention for children with MIH (Lygidakis et al. 2022).

The OHRQoL and well-being of children with MIH is a recent area of study. In contrast to a recent systematic review (Jälevik et al. 2022), our study found no difference in child-reported OHRQoL of children with MIH and those without. In Dublin, parents perceived the OHRQoL of their children with MIH to be poorer than parents of children without MIH. Despite a similar prevalence of MIH in both regions, and the same validated and internationally accepted instruments used to assess OHRQoL, no association was found in Cork-Kerry. These regional variations in the impact of MIH on OHRQoL may be influenced by differences in cultural attitudes towards oral health, as well as varying parental expectations regarding dental aesthetics and function.

In this study, the diagnosis of MIH was based on examination of the first permanent molars using the EAPD judgement criteria (Weerheijm et al. 2003b). Although permanent incisors are often affected by the condition, their involvement was not a prerequisite for diagnosing MIH. Nevertheless, in both Dublin and Cork-Kerry, parents of children with MIH were more likely to have noticed marks on their child’s permanent incisors. Furthermore, in Cork-Kerry, parents of children with MIH were less likely to be happy with the colour of their child’s permanent incisors. These findings suggest that the distinctive enamel abnormalities associated with MIH were apparent to parents and that for some, MIH may have adversely affected parental satisfaction with the colour of their child’s permanent incisors. There are reports in the literature of concerns regarding tooth discolouration both amongst children with MIH (Fragelli et al. 2021) and amongst mothers of affected children (Leal et al. 2017).

This study was the first to measure the prevalence of MIH in the Republic of Ireland and contributes Irish data to the ongoing global research regarding the prevalence and aetiology of MIH. A strength of this study was the large sample size, with a representative sample of 2310 children examined for MIH. A further strength was the use of the EAPD judgement criteria for MIH at the recommended age of 8 years old (Weerheijm et al. 2003b). The findings represent two geographical regions in Ireland and may not be generalisable to other regions. However, the study included children living in Dublin, the most populous region of Ireland. Whilst the examiners underwent extensive training for the assessment of MIH, calibration examinations were not carried out, which could have affected the validity and reliability of MIH measurements. However, the dental examiners were trained and calibrated to distinguish between fluoride and non-fluoride enamel opacities. Therefore, the risk of misclassifying fluorosis as MIH in this predominantly fluoridated country was minimised. Whilst the limitations of retrospectively investigating factors associated with the aetiology of MIH have been highlighted (Lygidakis et al. 2022), this study represents a first step for MIH research in Ireland. High-quality prospective studies are required to provide much needed clarity regarding the aetiology of MIH. These studies could be designed to explore systemic, environmental, and genetic influences on MIH development, which would help inform the development of targeted prevention and intervention strategies.

## Conclusions

MIH prevalence in two regions of Ireland ranged from 9 to 11%. There is an urgent need to measure the prevalence and severity of MIH nationally using standardised diagnostic and epidemiological criteria. The high prevalence of MIH and the strong association between MIH and dental caries in the newly erupted first permanent molars indicate a need for early identification and provision of tailored preventive and treatment services for children with MIH, in line with international best practise.

## Supplementary Information

Below is the link to the electronic supplementary material.

Supplementary file1 (DOCX 41 KB)

Supplementary file2 (DOCX 45 KB)

Supplementary file3 (DOCX 22 KB)
