# Supplementary material for: Prevalence of molar incisor hypomineralisation and associated factors amongst 8-year-olds in Ireland
Source: Eur Arch Paediatr Dent. 2025 Apr 28;26(6):1095–106. doi: 10.1007/s40368-025-01033-6 (PMC12638329; doi:10.1007/s40368-025-01033-6)
Supplement: Supplementary file 2 — Supplementary file2 (DOCX 45 KB) [file 40368_2025_1033_MOESM2_ESM.docx]

Prevalence of molar incisor hypomineralisation and associated factors amongst 8-year-olds in Ireland. European Archives of Paediatric Dentistry.

FACCT work stream 2

FACCT work stream 1

ESM Fig. 1: Overview of the FACCT study showing measurement of MIH in 8-year-old children in Phase 2 2016-17

Dental Electronic Health Records

Clinical Data Extraction

Validation against epidemiologic data

Retrospective analysis

2000 to 2007, 2007 to 2013-14

Economic evaluation of CWF

Parent/caregiver & child: aesthetic acceptability of fluorosis and dental caries questionnaire

Focus group / interview about CWF, toothpaste recommendations and aesthetic perception of fluorosis and dental caries

**Phase 1: School Year 2013-14**

Sample: Age 5 & 12 years

Dublin and Cork-Kerry

Study information, consent and parental questionnaire issued through schools

**Age 5**

Clinical Exam (dental caries)

Height & Weight

Parent/caregiver questionnaire

**Age 12**

Clinical Exam (dental caries and fluorosis)

Oral photograph

Self- perception of enamel opacities

IOTN (aesthetic component)

Height & Weight

Child questionnaire

Parent/caregiver questionnaire

**Phase 2:**

**School Year 2016-17**

Sample: Age 8 years

Dublin and Cork-Kerry

**Age 8**

Clinical Exam (dental caries and fluorosis)

Oral photograph

**Molar Incisor Hypomineralisation**

Height & Weight

Parent/caregiver questionnaire

Child questionnaire

Sample of parents/caregivers of children with caries and/or fluorosis
