# Supplementary material for: Prevalence of molar incisor hypomineralisation and associated factors amongst 8-year-olds in Ireland
Source: Eur Arch Paediatr Dent. 2025 Apr 28;26(6):1095–106. doi: 10.1007/s40368-025-01033-6 (PMC12638329; doi:10.1007/s40368-025-01033-6)
Supplement: Supplementary file 3 — Supplementary file3 (DOCX 22 KB) [file 40368_2025_1033_MOESM3_ESM.docx]

| ESM Table 1: Results of logistic regression (unadjusted) and multivariable logistic regression (adjusted) analyses of potential aetiological factors for MIH | | | | | | |
| --- | --- | --- | --- | --- | --- | --- |
| **Demographics and Potential Aetiological Factors** | **Dublin** | | | **Cork-Kerry** | | |
|  | Odds Ratio (95% CI) | | | Odds Ratio (95% CI) | | |
|  | Unadjusted | Adjusted | p value | Unadjusted | Adjusted | p value |
| Gender |  |  |  |  |  |  |
| Female | 0.78 (0.50, 1.22) | 0.80 (0.49, 1.29) | 0.361 | 1.10 (0.78, 1.55) | 1.07 (0.74, 1.53) | 0.730 |
| Male | Reference |  |  |  |  |  |
| Age | 0.93 (0.49, 1.78) | 1.02 (0.51, 2.07) | 0.949 | 1.41 (0.88, 2.25) | 1.33 (0.80, 2.20) | 0.265 |
| Economic disadvantage^a^ |  |  |  |  |  |  |
| Medical Card Yes | 1.08 (0.66, 1.76) | 0.90 (0.49, 1.65) | 0.735 | 0.49 (0.30, 0.78) | 0.44 (0.26, 0.75) | 0.003* |
| Medical Card No | Reference |  |  |  |  |  |
| Fluoridation Status |  |  |  |  |  |  |
| Full-CWF | N/A | N/A |  | Reference |  |  |
| Part-CWF | N/A | N/A |  | 1.11 (0.65, 1.87) | 1.04 (0.60, 1.81) | 0.887 |
| No-CWF | N/A | N/A |  | 1.16 (0.75, 1.79) | 1.03 (0.65, 1.63) | 0.889 |
| Unknown | N/A | N/A |  | 1.48 (0.65, 3.38) | 1.66 (0.72, 3.87) | 0.237 |
| Mother’s education |  |  |  |  |  |  |
| Leaving certificate or less^b^ | 1.32 (0.81, 2.15) | 1.22 (0.65, 2.28) | 0.545 | 1.22 (0.83, 1.81) | 1.65 (1.04, 2.63) | 0.034* |
| Diploma/certificate or higher | Reference |  |  |  |  |  |
| Father’s education |  |  |  |  |  |  |
| Leaving certificate or less | 1.18 (0.72, 1.94) | 1.03 (0.56, 1.90) | 0.928 | 0.96 (0.66, 1.39) | 0.89 (0.58, 1.37) | 0.593 |
| Diploma/certificate or higher | Reference |  |  |  |  |  |
| Child very healthy, no problems |  |  |  |  |  |  |
| Yes | Reference |  |  |  |  |  |
| No | 2.50 (1.58, 3.94) | 2.57 (1.57, 4.21) | < 0.001* | 1.00 (0.68, 1.46) | 1.16 (0.78, 1.73) | 0.466 |
| ^a^ Medical card ownership indicates socio-economic disadvantage, ^b^ Attainment of Leaving Certificate indicates completion of second level education, * p < 0.05, CI Confidence Interval, N/A Not applicable | | | | | | |
